# Multispectral Detection of Commercial Unmanned Aerial Vehicles

**DOI:** 10.3390/s19071517

**Published:** 2019-03-28

**Authors:** Jan Farlik, Miroslav Kratky, Josef Casar, Vadim Stary

**Affiliations:** Department of Air Defence, Faculty of Military Technology, University of Defence, Kounicova 65, 662 10 Brno, Czech Republic; miroslav.kratky@unob.cz (M.K.); josef.casar@unob.cz (J.C.); vadim.stary@unob.cz (V.S.)

**Keywords:** air defense, reconnaissance, detection, unmanned aerial vehicle, air threat, terrorism, drone

## Abstract

The fight against unmanned vehicles is nothing new; however, especially with the arrival of new technologies that are easily accessible for the wider population, new problems are arising. The deployment of small unmanned aerial vehicles (UAVs) by paramilitary organizations during conflicts around the world has become a reality, non-lethal “paparazzi” actions have become a common practice, and it is only a matter of time until the population faces lethal attacks. The basic prerequisite for direct defense against attacking UAVs is their detection. The authors of this paper analysed the possibility of detecting flying aircraft in several different electro-magnetic spectrum bands. Firstly, methods based on calculations and simulations were chosen, and experiments in laboratories and measurements of the exterior were subsequently performed. As a result, values of the radar cross section (RCS), the noise level, the surface temperature, and optical as well as acoustic traces of tested devices were quantified. The outputs obtained from calculated, simulated, and experimentally detected values were found via UAV detection distances using specific sensors working in corresponding parts of the frequency spectrum.

## 1. Introduction

Technological development connected with unmanned systems (unmanned aerial systems (UASs), unmanned aerial vehicles (UAVs), or simply “drones”) has seen a significant boom in the last ten years. Commercial unmanned flying has become a financially accessible leisure activity and hobby for thousands of people. The producers of commercial drones compete for potential customers, and they are constantly innovating and improving their products. These innovations continuously improve flight parameters such as flying range, speed, load capacity, and autonomous activity algorithms or artificial intelligence capable of transporting the drone through a number of obstacles [1]. Like any technology, drones can be misused. The aim of this article is to inform researchers about the results of a study that examined the detectability of commercially available drones. The goal is also to highlight specific results and implications from a series of measurements that were performed in the field of UAS detection in the period of 2014–2018. The authors of the study want to contribute to a greater awareness of drone misuse and offer defense options. The results and conclusions of the study are intended for researchers who are looking for specific defensive and protective solutions against drones.

As drones became available to the broad masses of population, they became available to criminal and terrorist entities (individuals and organizations) [2]. Today, unmanned vehicles, which can also be classified as low-flying, small, and slow (LSS) or low-flying, small, and fast (LSF), undoubtedly have a place in the field of airborne attacks [3]. Governments aiming to protect their citizens from the consequences of misuse have to deal with this. This is due to the continuous development and innovation in the field of commercial air vehicles and the constant struggle between potential aggressors and victims. However, the victims of UAV misuse are not limited to government institutions or infrastructure and are more often individuals or companies. These private entities need to be protected as well.

A separate issue is the deployment of drones in armed conflicts [4]—whether in the form of “regular” combat operations of regular armies or in asymmetric (or hybrid) conflicts, which are becoming more common nowadays [5,6]. The latter refers to deployment by paramilitary and guerrilla forces of paramilitary organizations.

The high quality of commercially accessible unmanned vehicles has the biggest influence on the potential risk of their misuse. There is almost unlimited obtainability, and the cost is low. Another risk is activities related to technological development that could, after implementation on a UAS platform, inflict a much greater threat than we can currently see. Many scientific teams make defense even more difficult as the resilience of UAS platforms is improved [7,8]. Nobody can be blamed for this fact, but it is necessary to point it out and to create defensive concepts that can cope with potential misuse.

The risk of successful UAS misuse is considerably increasing, and defense effectivity is decreasing, for example, in the following ways:

- A UAS is able to fulfil its task through autonomous flight without GPS [9,10];
- A swarm of UAVs can attack a target [11,12];
- A UAS is able to recognize a victim’s face and can target crowds [13,14];
- UASs have more remote control channels [15] and remote control options [16,17];
- UASs can be used to paralyze civilian air traffic control [18].

The miniaturization and the capability of operating for longer periods (thanks to batteries with a greater capacity), along with the implementation of other advanced technologies, enable UAS misuse. Therefore, defense will increasingly depend on the ability to effectively detect potentially rogue UASs and to lethally or nonlethally eliminate them [19]. We use the term “nonlethal elimination” to refer to ways in which one can take control of a drone and force it to land or when the drone’s on-board system is so jammed that it is not able to continue flight or fulfil its task. The term “lethal elimination” refers to destructive ways, such as shooting or the use of a directed-energy weapon (DEW).

There are several areas connected with countering UASs that can be summarized in the military term “air defense.” This purely military term is now used in connection with counteractions against commercially available UASs, which can, in the hands of individuals or groups, cause considerable damage. A drone does not have to be involved in a direct attack with the aim to kill people—the recent series of incidents at London’s Gatwick Airport show this [20]. The resulting failure of public transport leads not only to enormous economic losses, but also to indirect impacts, for example, on the confidence of the population in the functioning of developed industrial society itself. Moreover, the number of attacks is rapidly increasing [21].

According to the authors of the study, air defense that can be used for civilian purposes, or more precisely counter UAS (C-UAS), can include the following activities:

- The evaluation of threats and risks for a person, company, infrastructure, or state interests and the anticipation of vulnerability (Long-Term Threat Assessment and Anticipation);
- Infrastructure adjustment against unwanted UAS operations (Infrastructure Assessment);
- Surveillance of the surrounding area or eventually of the entire airspace and UAS detection (Surveillance);
- Acquisition of additional UAS data during flight and risk assessment of its behaviour (Tracking and Imminent Threat Assessment) [22];
- Rogue UAS identification and elimination orders (UAS Target Engagement);
- Continuous attempts, if technology allows, to detect and capture the operator (UAS Operator Detection and Capturing);
- Initiation of destructive (lethal) or non-destructive (nonlethal) actions against UASs to prevent the intentions of rogue drones;
- Assessment of counteractions against UASs;
- Improving measures to counter UASs.

Apart from technical detection methods, this study also focused on testing the ability to detect a UAS using basic human senses, i.e., eyes and ears. The reason for this is that human senses and vigilance will often play an important role during the final detection of incoming drones or as they fly over a target area.

This article presents findings from the field of small UAV detection. Other sequences of the engagement process, i.e., processing and elimination, are also briefly discussed. This study also summarizes the findings of four years of research in the field of defense against UASs and basic UAS detection parameters. The findings of the study confirm hypotheses regarding the abilities to detect small drones.

For brevity, we present a minimum amount of mathematical apparatuses on which the theoretical premises and simulations are based.

## 2. Materials and Methods

Typical drones of specific categories were selected to be tested for the experiments. Drones from mini and micro categories were chosen. These UAVs are characterized by their small dimensions, their radiated heat, and a very small RCS. These attributes make their detection a difficult task, requiring a good means of detection and trained operators.

The nano category (according to Table 1) was not involved in the study due to commercial unavailability and to their short operational range at the time of its creation (we ignored the part of the market dealing with toy drones). Drones from the nano category were assumed to be less effective.

Drones were tested in several electromagnetic spectrum bands in accordance with [7]: radar (RL), radio-frequency (RF), electro-optical (EO), infrared (IR), and acoustic. In some cases, drones were tested in a multispectral field through “combined sensors.”

**Figure 1:**
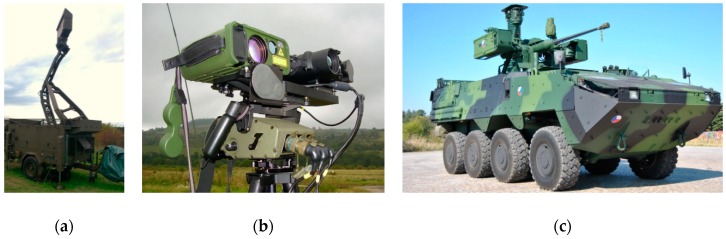
(**a**) ReVisor radar, (**b**) Recco apparatuses ReTOB Retia, and (**c**) Pandur vehicle sensors.

The anechoic chamber and the laboratory at the University of Defence (Czech Republic) were used to perform internal experiments. The following military technologies were used to perform external experiments (see Figure 1):

- ReVisor radar of Retia Inc. (Pardubice, Czech Republic)
- The air observer apparatus ReTOB of Retia Inc. (visual, infra, laser range-finder);
- Other experimental sensors at the University of Defence, Retia Inc. and Dedrone company (San Francisco, CA, USA).

The cooperating companies for the experiments were Retia Inc. [CZ], 602 LTD. [CZ], DeDrone Inc. [USA], a commercial CZ partner, Jamcopters LTD [CZ], and the VTU state comp [CZ].

### 2.1. Selected UAVs

**Figure 2:**
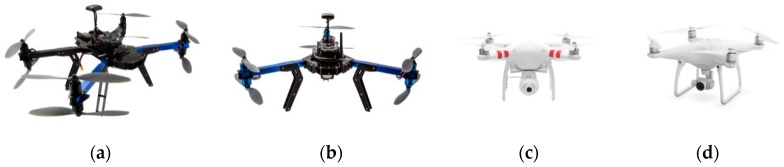
Selected drones: (**a**) 3DR X8; (**b**) 3DR Y6; (**c**) DJI PHANTOM 2; (**d**) DJI PHATNOM 4.

These five drones were selected for internal experiments (Figure 2 depicts just four of them):

- Octocopter 3D Robotics (3DR) X8 (Figure 2a);
- Quadcopter DJI PHANTOM 2 Vision (Figure 2c);
- Quadcopter DJI Phantom 4 (Figure 2d);
- Hexacopter: 3D Robotics (3DR) Y6 (Figure 2b);
- Quadcopter DJI MAVIC Pro.

**Figure 3:**
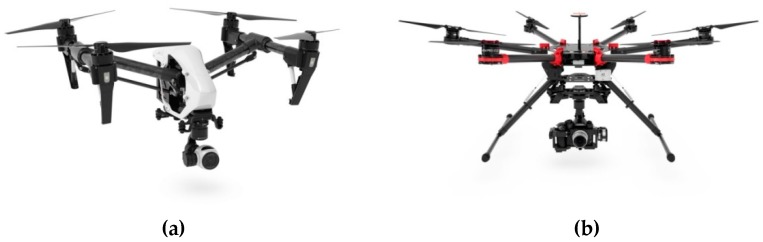
Selected drones: (**a**) DJI Inspire 1; (**b**) DJI Spreading Wings S900.

In addition to those mentioned above, the following drones were selected for external experiments:

- Quadcopter DJI Inspire 1 (Figure 3a);
- Hexacopter DJI Spreading Wings S900 (Figure 3b), S800EVO;
- Quadcopter TAROT F650.

These drones were selected based on a sales analysis of Amazon and Techradar and on a flight parameter analysis, considering the flight time (operating time), the load capacity, and the teleoperation capability. The X8, Y6, and S900 drones are professional UASs, unlike the other three, which are better classified as hobby drones.

### 2.2. Methods and Procedures Used During Experiments

**Figure 4:**
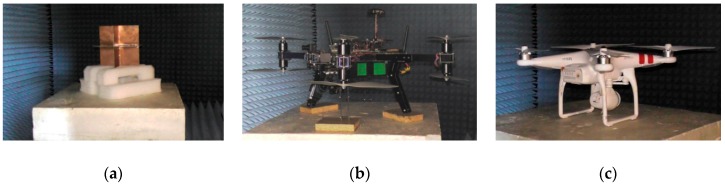
Anechoic chamber experiment: (**a**) corner-cube reflector; (**b**) 3DR X8; (**c**) DJI PHATNOM 2.

The procedures within the internal experiments, using the anechoic chamber and other laboratories, were as follows:

- Installation of the workplace—the anechoic chamber with accessories;
- The calibration of the anechoic chamber without samples;
- The calibration of the anechoic chamber using a corner-cube reflector (Figure 4a);
- Phased measurement of samples (Figure 4b,c);
- Data processing using the software of the anechoic chamber;
- Data processing using Matlab software;
- Evaluation of the measurement according to the technical aspects;
- Conclusion processing and evaluation from an operational point of view.

**Figure 5:**
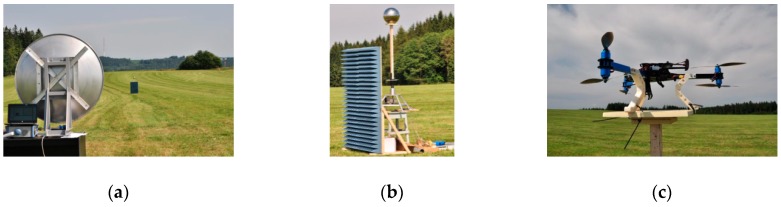
External experiment: (**a**) parabolic radar antenna; (**b**) sphere radar reflector; (**c**) 3DR Y6.

The procedure of external experiments for RCS measurement using radars included the following:

- Experiment preparation and measurement design;
- Workplace set up in the terrain (Figure 5a);
- Rectification of the antenna system in the direction of the stand, which is designed for fixing the measured samples, to prevent the potential detection of side loops of the measuring antenna;
- Measurements of the terrain reflections without measured samples (the background) and the detection of natural objects in the space (clutter); “remote gate” setting for spatial selection of the base with samples;
- Calibration by means of reference sample measurements:
  ○ a sphere (Figure 5b);
  ○ a corner-cube reflector of 0.1, 0.3 and 0.5 m^2^;
- Drone (Figure 5c) and payload measurement (10 × 10 cm metal cube model);
- An evaluation of experiments in terrain conditions;
- An evaluation of the experiment in laboratory conditions including directional diagrams;
- Conclusions and evaluation from an operational point of view.

**Figure 6:**
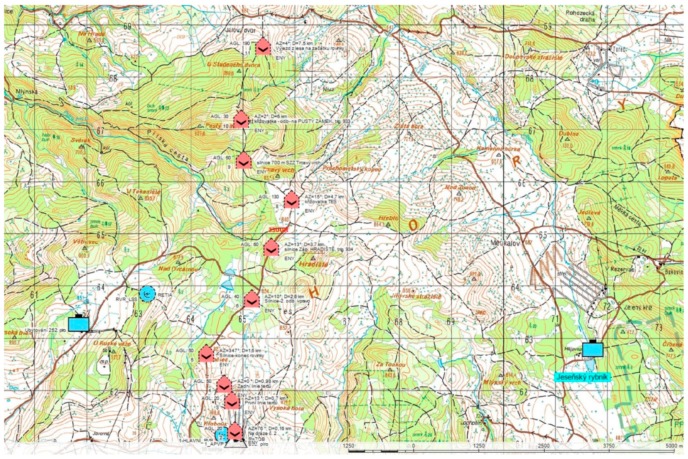
External experiment scenario: detectability, and UAV (red) and observer post (blue) positions.

In external experiments, sensors listed at the beginning of Section 2 were used. The process of external experiments using visual methods of detection included the following:

- Preparation of the measurement area and identification of the observation points and the UAS operations sites (Figure 6);
- Installation of three types of visual observers:
  ○ a visual observer without a device (unarmed eye);
  ○ a visual observer with a telescope;
  ○ a visual observer with a special device (visual, IR, laser range finder);
- UAS control flights (successively from the posts closest to the observer);
- Measurement of UAS visibility and audibility;
- Result evaluation;
- Processing of the result of the experiment.

## 3. Radar and Radio-Frequency Methods for UAV Detection

The experiments in the radar spectrum were preceded by an analysis of individual frequencies suitable for the detection of small-sized targets. Due to the small RCS, the X band was chosen, which is widely used with radars with a shorter range (up to 100 km), such as those used in this study.

The first phase of experiments included the simulation of objects and their RCS in SuperNEC software. The software was used for the 3D modeling of objects and their subsequent analysis from a theoretical RCS point of view (see Calculations and Simulations).

In the second phase of the experiments, the available drones were measured in the anechoic chamber under ideal conditions (see Laboratory RCS Measurement), and external experiments were performed using experimental and commercially available radars (see Field Experiments of RL Detectability). Some data concerning characteristics and frequencies cannot be disclosed due to their sensitivity.

Important aspects of target detection (in the RL spectrum) are the target characteristics. These characteristics are important because the signal reflected from the target is altered before returning to the radar receiver. Changes are caused by the characteristics of the captured object (target). The reflection of a target depends on a number of factors, of which the most important are the material used, the shape of the target, the relation between the dimensions and the wavelength used, the wave polarization. The reflexion capability of the target was evaluated using RCS due to the impossibility of finding the exact impact of these factors. The radar cross section is considered isotropic and non-absorbing, but it is an unreal surface and only indicates the amount of reflected power towards the radar.

### 3.1. Calculations and Simulations

**Figure 7:**
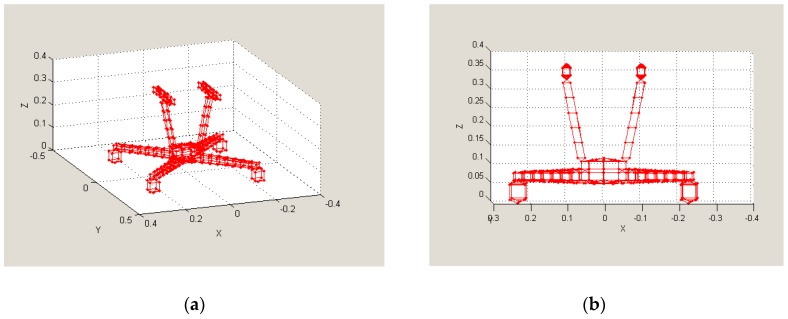
Quadcopter model in SuperNEC SW [23]: (**a**) 3D view; (**b**) X-Z view.

**Figure 8:**
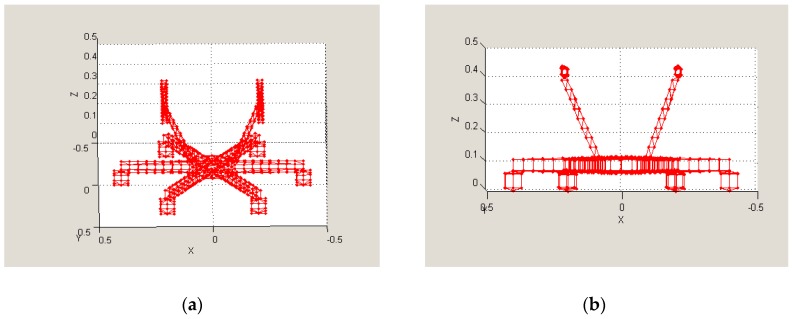
Hexacopter model in SuperNEC SW [23]: (**a**) 3D view; (**b**) X-Z view.

The aim was to create computer models of selected unmanned vehicles and to calculate their RCS using software tools. The models representing both the quadcopter (Figure 7) and the hexacopter (Figure 8) were created (in “SIG GUI” of SuperNEC software), and they were both subsequently augmented by an object representing the possible payload. The calculations were based on the SuperNEC software, which is not intended to directly determine the RCS, but to record and express the radiated power of the irradiated object. It can be accepted for experimental purposes. The object was irradiated by a constant wave, and its parameters were chosen by the operators of the simulation, depending on experiment requirements.

However, the model design revealed, in the final phase, minor deviations from the real copter shapes. These problems were caused by the fact that the SuperNEC GUI software is not intended to design such small-sized objects.

**Figure 9:**
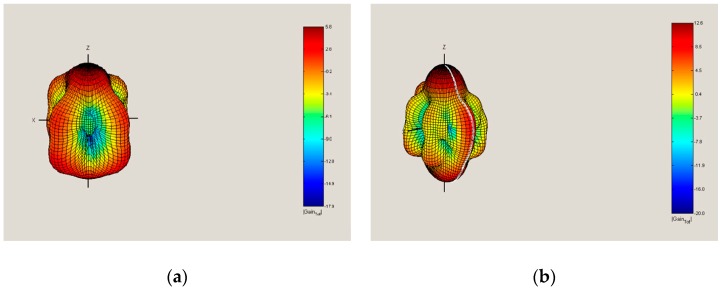
Power gain [23]: (**a**) Quadcopter; (**b**) Hexacopter.

Subsequently, a simulation was created, where the results of radiated power were produced and consequent RCS determination of each device was performed. Furthermore, it was necessary to ensure the selection of suitable materials for different parts of the drone from an electrical conductivity perspective to achieve the most accurate results. Material from which the drones are composed, mostly plastics and metals, was chosen. The chosen material for the engine and motherboard, where all the avionics are placed, was copper. The rest of the material used was handled as an electrical insulator. The next step was to set the number of radiators to a value of 3 (providing omnidirectional radiation) and to determine the distances between the signal emitters and the irradiated vehicles at a distance of 3 m, which corresponds to the subsequent RCS measurement in the anechoic chamber, where only one emitter was used. The output from the simulations was the omnidirectional power gain, from which the RCS of all vehicles was subsequently determined. The calculation of the RCS was determined in the first case from the omnidirectional power gain and in the second case from the gain in the horizontal plane directly at the level of the signal emitter (Figure 9). Results again corresponded to the measurements in the anechoic chamber.

All calculated values were exported to the MATLAB computational mathematical log. Table 2 provides the measured values for specific frequencies.

Disclaimer to Table 2: Table 2 shows, in some cases, higher RCS values for lower frequencies in comparison with smaller RCS values for higher frequencies. This may be caused by imperfect modelling of the UAV in the SuperNEC SW, because there are limitations in modelling small parts. The authors suppose that real experiments, described in this paper, proved statements better.

The calculation of individual RCSs was performed for different frequency bands according to the IEEE international table. Six simulations for all the created models were performed with different frequencies in L, S, C, and X bands. The RCS had to be calculated right after the simulation because the output of the simulation was limited to power gain, not the real RCS. Therefore, it was necessary, for all frequencies, to perform a simulation of the reference object, namely a sphere with radius of r = 0.5 m, for which the reflecting area could be theoretically calculated and the conversion coefficient could be subsequently determined. According to the assumptions and considering the results, it was evident that using a higher frequency of signal, in the X-band, is the best way to detect these kinds of UAVs.

### 3.2. Laboratory Evaluation of RCS

Laboratory measurements were performed in the anechoic chamber (as mentioned above). At first, it was necessary to calibrate the measurement and the coefficient of conversion between the received power [dBm] and the RCS [m^2^]. The reference object used for the subsequent calculation of the conversion coefficient was a metal cube with an edge of d = 0.1 m. All measurements were performed using a constant frequency signal of f = 10 GHz. The theoretical value of the copper cube was RCS_dmax_ = 1.39 m^2^.

**Figure 10:**
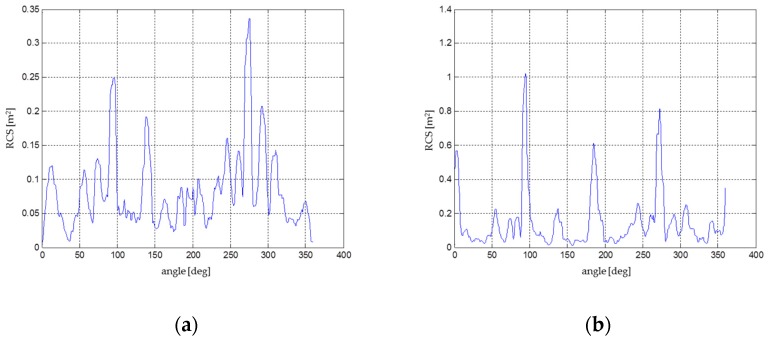
RCS [23]: (**a**) Quadcopter; (**b**) Quadcopter with Corner-Cube Reflector.

**Figure 11:**
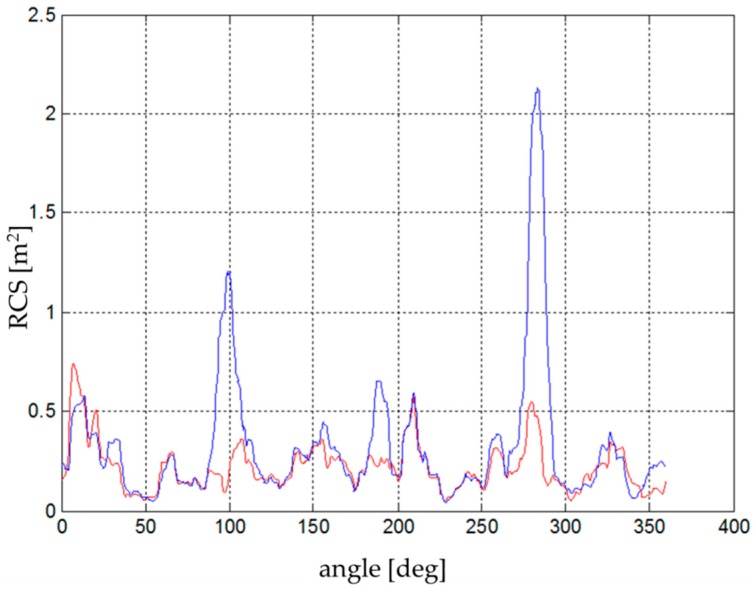
RCS of Hexacopter (red) with Corner-Cube Reflector (Blue) [23].

As in the previous experiment—the software simulation—four different measurements were performed in total. The tested copters were gradually irradiated, followed by the copters with a corner-cube reflector attached (Figure 10 and Figure 11). Acquired results of the individual power gains were converted to an RCS using the previously calculated conversion coefficient.

### 3.3. Terrain Experiments of RL Detection

This chapter contains the RCS measurement results of selected drones (DJI Phantom 2, DJI Phantom 4, 3D Robotics Y6, and 3D Robotics X8). All measured RCS data were converted to square meters for improved legibility. The experiment was performed in close cooperation with Mr. Sedivy (Retia Inc.) (see Acknowledgement).

As a comparison, the B-52 bomber had a 100 m^2^ RCS, the Mig-21 fighter aircraft had an approximately 3 m^2^ RCS, the F-18 had an approximately 1 m^2^ RCS, and the Tomahawk missile had an approximately 0.5 m^2^ RCS.

**Figure 12:**
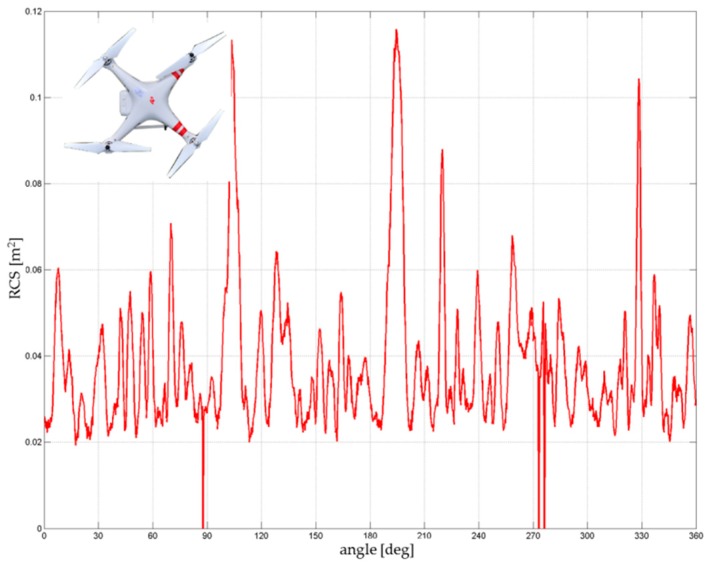
RCS directional diagram: DJI Phantom 2; frequency range: 8.6–9.4 GHz.

DJI Phantom 2 had a common RCS in the range of 0.02–0.06 m^2^ (Figure 12). The calculated average was approximately 0.033–0.038 m^2^. Significant peaks with values above 0.1 m^2^ were caused by the four engines and especially by the mounted camera and the internal arrangement of the electronics. These peaks, however, cannot be considered because the drone in real flight operation was rarely turned to the sensor at these maximum edges. The important range, in terms of the threat of the approaching drone (the frontal flight of the attacking drone to the sensor), was approximately from +/− 20° to 30°.

**Figure 13:**
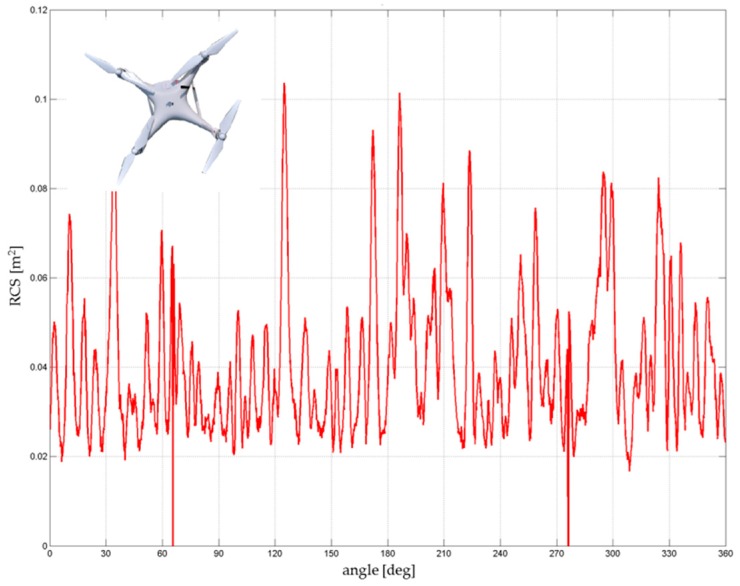
RCS directional diagram: DJI Phantom 4; frequency range: 8.6–9.4 GHz.

DJI Phantom 4 and its predecessor DJI Phantom 2 have similar RCS characteristics due to their almost identical construction characteristics (Figure 13). Considering the more noticeable peaks above 0.06 m^2^, the average RCS of the drone was increased by about 0.002 m^2^ to the resulting 0.035–0.04 m^2^. Obviously the RCS slightly increased due to improved flight characteristics (a higher weight of battery, on-board electronics, and the camera).

**Figure 14:**
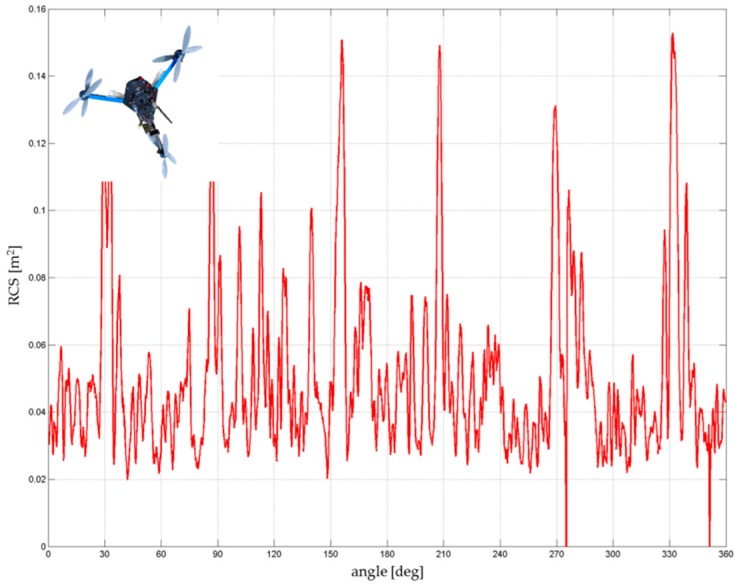
RCS directional diagram: 3DR Y6; frequency range: 8.6–9.4 GHz.

Regarding the 3D Robotics Y6, there are six peaks in the diagram (Figure 14) where the RCS exceeds the value 0.1 m^2^, caused by the three metallic arms. These peaks are repeated regularly after approximately 60°, which confirms the influence of the robust arms. It is to be assumed that the probability of detection of this kind of drone (if compared with DJI drones) will be higher. The average RCS is close to 0.05 m^2^, which significantly increases the distance of detection.

**Figure 15:**
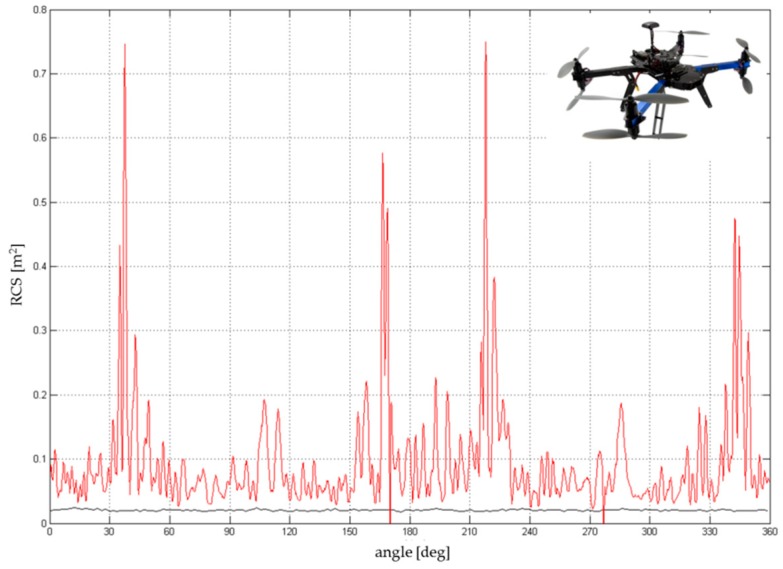
RCS directional diagram: 3DR X8; frequency range: 8.6–9.4 GHz.

The 3D Robotics X8, constructed with eight coaxial rotors, is composed of four individual arms with two rotors on each arm. The largest RCS corresponds to the position of the arm with respect to the sensor, and it is also shifted by 90°. Different RCS peaks and eccentricity were determined by the construction and asymmetry of the on-board electronics. Due to a higher amount of body mass and materials (metal), the total RCS was higher than that of the 3DR Y6 and fluctuated between 0.05 and 0.15 m^2^. The average RCS was in the range 0.07–0.1 m^2^ (Figure 15), but some of the peaks reached values up to 0.75 m^2^ in the case of a random sum of several components.

Based on the RCS measurements, commercial UAVs (similar to DJI Phantom 2, 3, or 4) equipped with an on-board camera and other optional devices (e.g., an Improvised Explosive Device (IED) sensor), where the overall weight was about 0.5 kg, had an average RCS of 0.04–0.05 m^2^.

### 3.4. Radio-Frequency Detection Experiments

Radio-frequency analysis of UAS signals is common in UAS detection. The principle is based on the evaluation of the signal transmitted by the control station (RC controller signals—“uplink”) or the signal transmitted by the UAV (“downlink”), e.g., telemetry data and video signals.

Popular commercial drones use WiFi frequencies (2.4 or 5.8 GHz). In accordance with national frequency allocation tables (e.g., in Czech Republic, that of the National Radio Frequency Agency—NARFA), other frequencies can also be used, e.g., UHF 433 or 434 MHz. The maximum output power of a transmitted signal is also regulated by national laws.

One of the most important aspects in the detection and identification of UAS is the data protocol used. RF detectors are designed based on knowledge of the system topology and structure, primarily knowledge of the transport layer. For precise identification, a database (library) of UAV frequencies and protocols is needed. A library allows a user to detect and identify the airborne target (drone) “online” based on its data signals. The disadvantage of this approach is the possibility of only detecting and identifying the drones with known parameters (which are included in the database). Detection of non-standard drones in urban environments may not be successful.

The main aim of the experiment was to evaluate the abilities and the possibility of RF drone detection by commercial RF detection devices using allowed frequencies.

**Figure 16:**
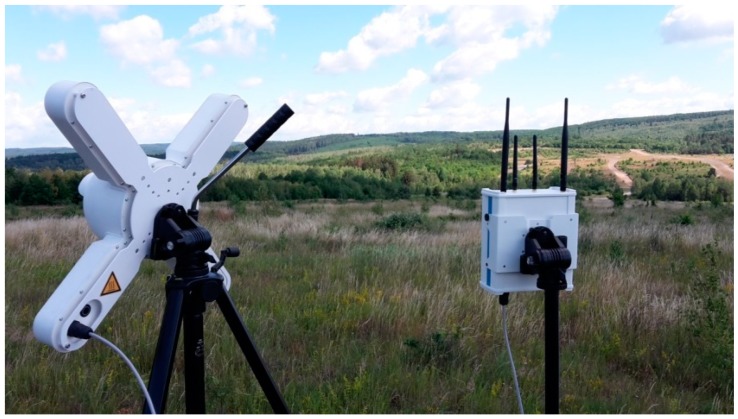
Dedrone Tracker Event Kit.

We used an RF detection device produced by Dedrone Inc. [USA & Germany], the DroneTracker Event Kit in Demo configuration; this kit consists of a Drone Tracker Multi Sensor and a DroneTracker RF Sensor (Figure 16). The multi sensor contains an RGB camera and a pair of microphones. The experiment took place on slightly hilly terrain with occasional trees and no buildings. The detector was placed in a spot with a direct view of the UAV flight route and the RC controller position.

The reference UAVs for this experiment were DJI MAVIC Pro, DJI Phantom II, and 3D Robotics Y6 (Arducopter). Measurement was performed at distances from 70 m to 1.7 km, and successful/unsuccessful detection and identification were evaluated.

In the given environment conditions, it was possible to detect (the Dedrone system reported “ALARM”) and identify (the Dedrone system reported “UAV”) the UAV 3D Robotics Y6, which uses 2.4 GHz RC (Spectrum DX7) and 433 MHz telemetry, connected through the MAVLink protocol. The distance was close to 2000 m (see Table 3). In the case of the UAV DJI MAVIC Pro, the system was able to detect the UAV at a distance close to 1500 m (no identification, just detection) for more than 50% of the UAV flight time. However, the detection was unstable, so the identification was not possible. The table below shows the measured data (*A* = Alarm; *I* = Identification).

## 4. Infra-Red, Optical, and Acoustic Methods of UAV Detection

The following methods are based on other characteristic attributes of the UAV. These methods use other segments of the electromagnetic spectrum or acoustic signals and can be defined as “passive” methods of detection. A major benefit of these methods is fewer technical requirements. However, prepared operators and a quick evaluation of the acquired data are still essential. Sets of experiments was performed in the laboratory and in a real environment.

### 4.1. Laboratory Experiments of Infra-Red (IR) Detectability

The infrared spectrum is part of the electromagnetic spectrum, with a wavelength from 760 nm to 1000 μm. The surveillance of the aerial area in the IR domain is mainly used for the detection of low altitude aerial targets, and it is part of an additional surveillance method whereby “blind” areas in the local air picture (LAP) are covered. The advantage of this type of detection is its independence from the RCS, but there is a limitation based on the physical principles and the dependence on the environment, so the effective range of this method is usually smaller in comparison with detection in radar spectra.

The experiment made use of a thermal imaging camera (FLIR A40M) connected to a PC and of appropriate control and evaluation software. For the evaluation of the thermal image from the camera, ThermoCAM Researcher Professional 2.9, ThermoCAM Explorer 9.9, and MATLAB r2011a were used. The environmental conditions in the laboratory consisted of a temperature of 21 °C temperature, a humidity of 40%, and no air motion.

The experiment consisted of two parts. The first part was focused on the measurement of a thermal image of the isolated electric motor (850Kv AC2830-358, jDrones, Bangkok Thailand), and the second part was focused on a thermal image of the entire UAV.

First, the contrast of the thermal sign of the motor and the UAV in the background was evaluated using the emissivity value. The emissivity is defined as the ratio between object radiation and the radiation of heat from an ideal “black body” with an emissivity coefficient of ε = 1. It generally depends on several parameters, such as object temperature, angle of view, wavelength, the colour and structure of the object surface, and object distance [24].

**Figure 17:**
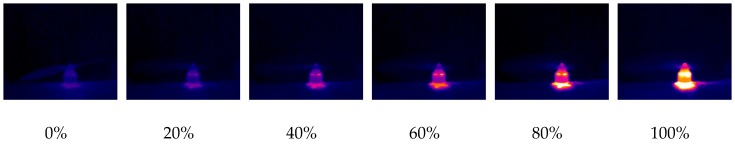
Thermal image of the electric motor: output power (RPM): 0–100%.

It is assumed that the temperature of the electric motor (UAV) depends on the input power. Based on this dependency, the theoretical distance of detectability was calculated and compared with the measurement results in the real environment. The isolated electric motor was mounted on a static platform and measured by the thermal imaging camera (Figure 17). The motor power and therefore the speed (in rounds per minute—RPM) was controlled by the Arduino UNO microcontroller software [25].

**Figure 18:**
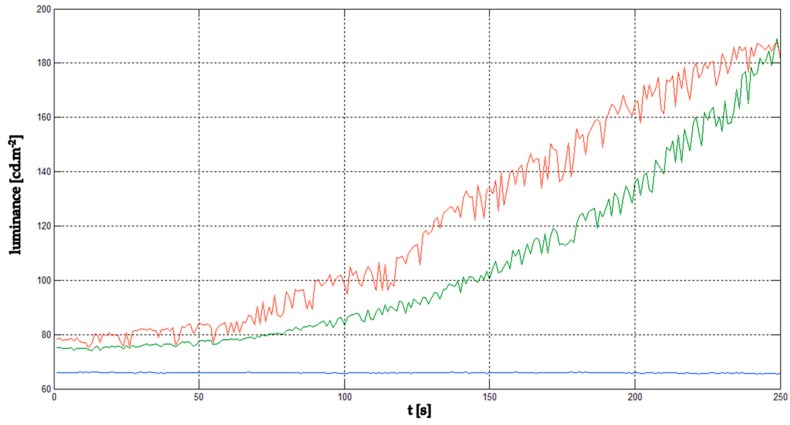
Luminance graph of the motor: maximum (red), minimum (green), and background (blue).

**Figure 19:**
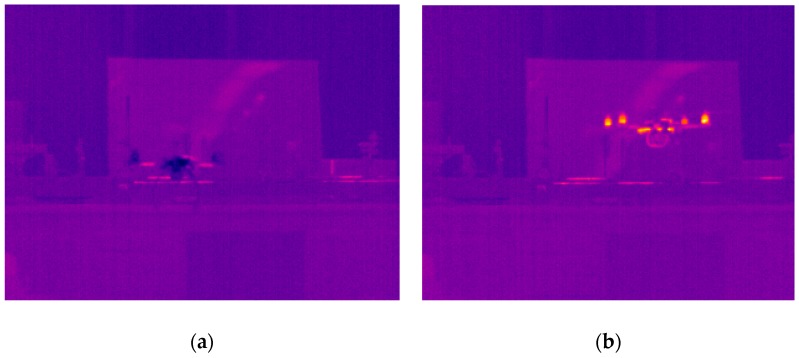
Thermal image of quadcopter: (**a**) beginning of measurement; (**b**) after 140 s.

**Figure 20:**
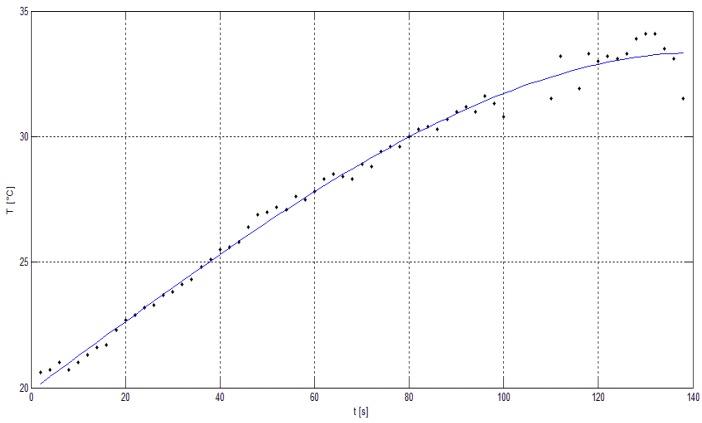
Electric motor temperature graph: quadcopter (0–140 s).

**Figure 21:**
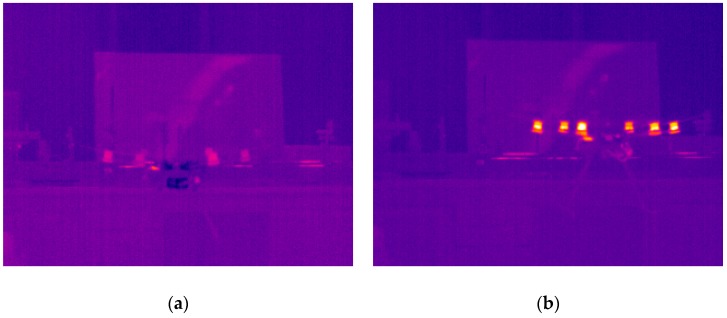
Thermal image of hexacopter: (**a**) beginning of measurement; (**b**) after 140 s.

**Figure 22:**
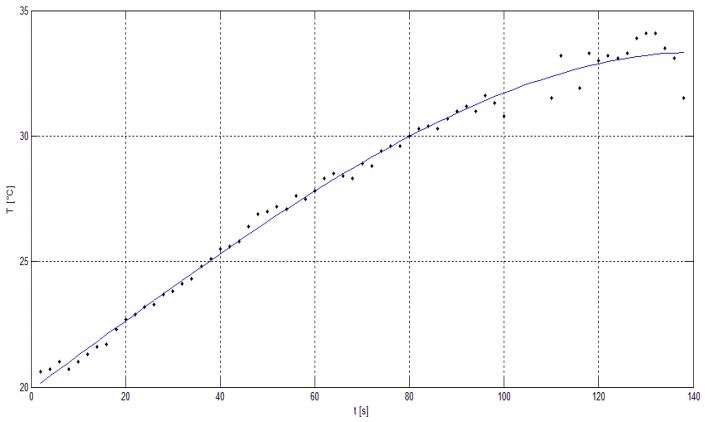
Electric motor temperature graph: hexacopter (0–140 s).

The next part of the experiment was focused on the evaluation of the thermal image of the whole UAV during the flight. The hexacopter DJI S900 and quadcopter TAROT F650 were used as reference samples. It was critical to keep the position of the UAV in the field of view of the thermal camera. For this reason, the total power of the electric motors and their RPM values depended on the UAV position and could not be adjusted in the way they could be in the case of an isolated electric motor. Based on these limitations, the luminance dependence on time was measured (Figure 18). Figure 19 and Figure 20 show the output from the thermal camera and motor heating process of the quadcopter and Figure 21 and Figure 22 represent the outputs of the hexacopter, both for a distance of 8 m between the camera and UAV.

The experimental results indicate the possibility of UAV detection in the IR part of the electromagnetic spectrum. The thermal radiation of the different parts of the UAV, mainly the electric engines, is evident. The average temperature difference between the beginning of the experiment and the final temperature (after 150 s) is 12 °C for the quadcopter and 18 °C for the hexacopter. Detection, recognition, and identification were determined based on these results and the STANAG 4347 [26]. The simplified Johnson criteria were applied: the term “detection” means that some entity—not noise—is in the field of interest; the term “recognition” means the object is likely a small, multirotor UAV; and the term “identification” means that this object is a quadcopter UAV (it could be a DJI product and is probably of the TAROT F### series).

The limit of UAV TAROT F650 detection, recognition, and identification is 300, 70, and 30 m, respectively, in the laboratory environment with the FLIR A40M thermal imaging camera. The images acquired in the IR spectrum were used for the following image processing.

### 4.2. Experiments of Optical and Acoustic Detectability in a Real Environment

The UAV detectability results from the laboratory experiments were compared with results from a series of real environment (terrain) measurements. This set of experiments was performed between 2015 and 2018 (see Section 4.2.4). The aim of these experiments was to evaluate audibility and visibility by human senses and with established technical devices for optical and IR detection.

#### 4.2.1. Visible Optical Spectrum

Elementary visual detection of the UAV can be done by human sight or with optical devices. Analysis of the human sight detection method is described in Section 4.2.3 in more detail. The main point of the experiments with the optical devices (commercial and military optical apparatuses, e.g., binoculars and cameras) was to evaluate the detection and tracking of UAVs in a real environment.

**Figure 23:**
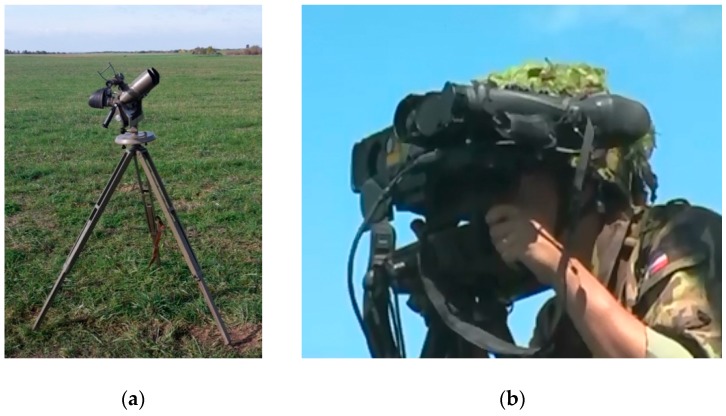
Optical devices: (**a**) TZK binoculars; (**b**) an ReTOB air observer device.

List of evaluated technical devices:

- ReTOB (RETIA co.)—a military air observer device, maximal optical zoom—8× [27];
- LEICA GEOVID 7×42 BD—binoculars, maximal optical zoom 7× [28];
- NIKON D5100—a digital camera, maximal optical zoom 6× [29];
- PANASONIC HC-WX979—a digital 4K camera, maximal optical zoom 20× [30];
- TZK – air defense military binoculars, maximal optical zoom 10× [31]. See Figure 23.

The series of experiments in the real environment were performed using the above-mentioned technical devices. The experiment configuration and conditions were as follows:

- climate zone and location: temperate, Czech Republic;
- weather and temperature: sunny, 15 °C (measured by Extech EN300);
- visibility: 30+ km;
- wind speed: 0–9 ms^-1^ (measured by Extech EN300);
- humidity: 45–50% (measured by Extech EN300);
- UAV types: DJI Phantom IV, DJI MAVIC Pro, DJI INSPIRE 1, and DJI MATRICE 600 (UAV dimensions cover the portfolio of the most common UAV types);
- UAV distance from the observer post: 0–1.3 km;
- UAV flight altitude: 100 m AGL;
- UAV flight speed: 10 ms^-1^;
- number of evaluators/observers: 3;
- level of observer skills:
  ○ beginner (researchers);
  ○ in case of the ReTOB, professional (an air observer crew was employed (Czech Army)).

**Figure 24:**
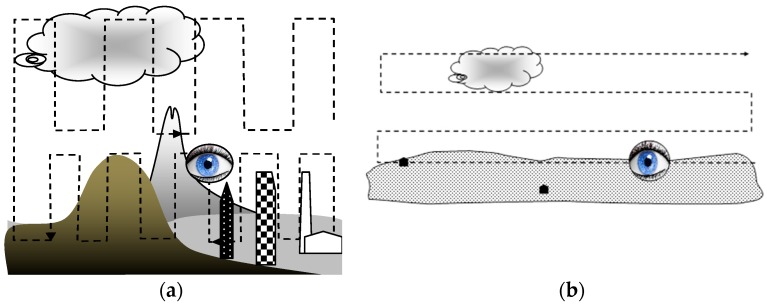
Observation methods: (**a**) difficult terrain, closer targets; (**b**) plain terrain, distant targets.

Figure 24 illustrates the air space observation methods for the different types of terrain at different distances for the LSS or LSF target categories.

The Experiment Results:

Generally, a micro/mini UAV is difficult to effectively detect visually, especially while in motion. The chance of visual detection without previous knowledge of the UAV position at distances above 100 m is very small; at distances above 300 m, it is almost impossible (depends on the dimensions of the UAV and the detection device).

**Binoculars LEICA 7×42**: With preliminary localization by human sight, detection was possible up to around 200 m. Tracking of the localized UAV without a tripod was possible at a distance up to 1.2 km. However, effective usage of the laser range meter was only possible with a tripod.

**TZK 10×80 air defense military binoculars**: With a known location, it was possible to detect the UAV with binoculars. After the field of view (FOV) was spread, it was possible to track the UAV by the main objective lens at a distance up to 1.2 km (in good viewing conditions, even more).

**PANASONIC HC-WX979**: Effective usage for real-time (“online”) detection was possible at a distance up to tens of meters because of an insufficient size and resolution of the camera display. Moreover, the tripod was not suitable for aerial object detection due to its limited angle range. However, it was possible to use the video for image processing and to apply computer vision algorithms.

**NIKON D5100**: This device was equipped with an optical viewfinder, but this was still not suitable for real-time detection because of the absence of optical zoom. It was possible to use the video data for image processing and apply computer vision algorithms.

**ReTOB**: This device attained the best results, mainly because it is a professional device for aerial target detection and it was used by skilled professionals. In the preliminary localization of the UAV (e.g., by the LAP from the radar), it was possible to detect and track the UAV at a distance >1.5 km.

Based on the experimental results and on the air defense operator training, we conclude that, with well trained and skilled operators, it is possible to increase the maximum distance of detection by up to 25%.

#### 4.2.2. Optical Infrared Spectrum

This set of experiments was performed in a real environment under low visibility conditions caused by the time of day and the atmospheric transmittance in the visible spectrum. The TAROT F650 quadcopter and the DJI S800E hexacopter were used to represent mini UAVs generally.

A professional military device for optical air surveillance, ReTOB, produced by the Retia company, was used. A MATIS HH thermal camera and a laser range finder, the main parts of the ReTOB device, were mounted on a tripod with a goniometer, and this device was connected to a command and control system via a radio station. The camera operated with a 3–5 µm wavelength and an angle of view of 9 × 6°.

The second tested device was an FLIR ThermoVision^TM^ A40M/Researcher thermal imaging camera, which uses a wavelength of 7.5–13 µm and an angle of view of 24 × 18°. With additional objective lenses, the FOV was from 7 × 5.3 to 80 × 60°.

**Figure 25:**
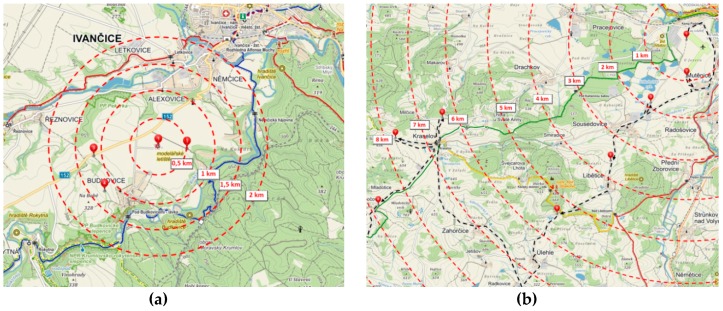
Scenario layout: (**a**) LKBUDK; (**b**) LKST.

The experiment took place in two areas: Area 1, the Budkovice airport [CZ] (ICAO: LKBUDK), and Area 2, the Strakonice airport [CZ] (ICAO: LKST). We followed a prepared scenario wherein the position of the UAV and the observer post was planned in advanced according to the scenario map (see Figure 25).

Detectability was evaluated first on a terrain background and then on a sky background. The flight direction was towards the observer. Accurate data of the UAV position were obtained from UAV telemetry and correlated with the actual situation.

**Figure 26:**
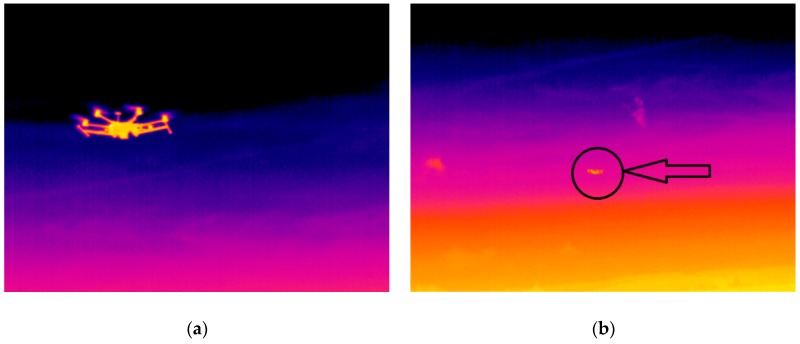
IR Image of the hexacopter from the FLIR A40M: (**a**) 10 m away; (**b**) 70 m away.

Data from the sensors and from the UAV were evaluated. After determining the UAV and observer positions, we obtained the following findings: The FLIR thermal imaging camera without an additional objective lens was able to detect both types of UAV at a maximum distance of 140 m. Recognition was possible at distances of 70 m (hexacopter) and 40 m (quadcopter), see Figure 26. With an additional objective lens, it was possible to extend the maximum detectability range, but the FOV was very narrow for effective usage (target tracking).

The MATIS HH thermal imaging camera yielded generally improved results. Effective detection of the UAV was possible at distances up to 1.8 km, recognition up to 1 km, and identification up to 0.4 km. However, the camera was subject to the same limitation of a narrow FOV. Therefore, it is necessary to have accurate preliminary information regarding the UAV direction.

#### 4.2.3. Acoustic Spectrum

Experiments with Acoustic Sensor Devices:

This set of experiments was performed in a real environment. The TAROT F650 quadcopter and the DJI S800E hexacopter were representatives of the mini UAV category. UAV noise was recorded. These data were post-processed and evaluated according to the noise level of the background.

The experimental condition was as follows:

- climate zone and place: temperate, Czech Republic;
- weather and temperature: sunny, 5 °C (measured by Extech EN300);
- wind speed: 4 ms^-1^ (measured by Extech EN300);
- humidity: 70% (measured by Extech EN300);
- background noise level: 55 dB (measured by SONY F-V120);
- UAV distance from the sensor post: 8 m;
- UAV flight altitude: 5 m AGL;
- flight method:
  ○ stabilized altitude hold (constant motor RPM);
  ○ maneuver (variable motor RPM).

**Figure 27:**
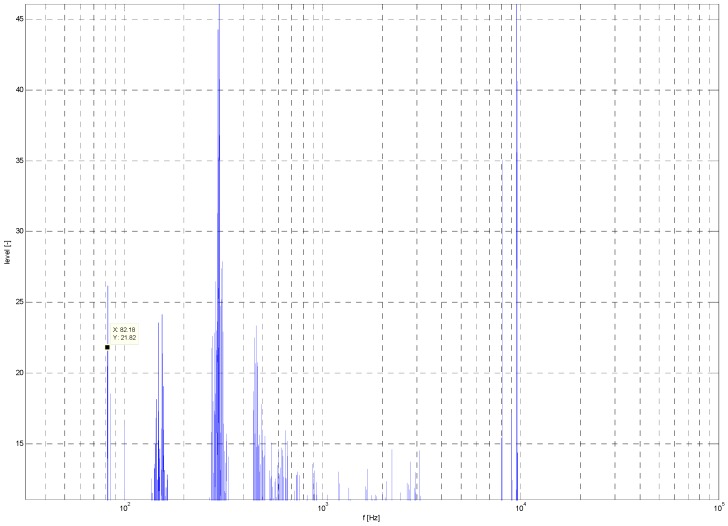
Quadcopter noise frequency spectrum.

**Figure 28:**
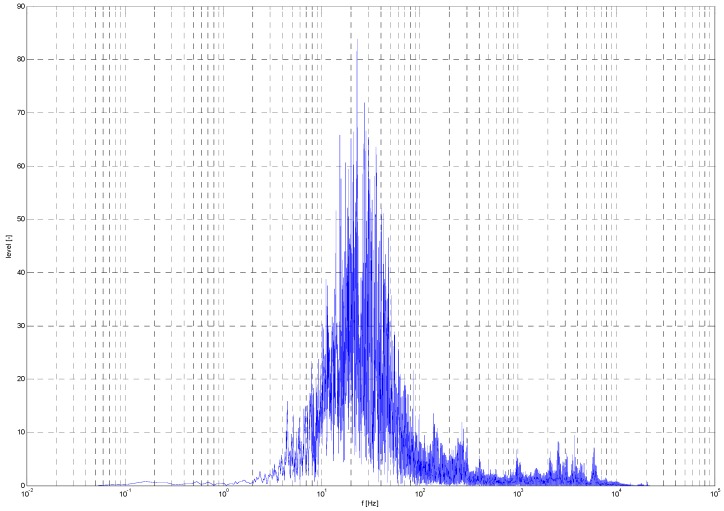
Hexacopter noise frequency spectrum.

Noise data were acquired by the SONY F-V120 microphone and post-processed in MATLAB r2011a. During post processing, data were transformed from time to frequency. The result is displayed as a frequency response chart (Figure 27 and Figure 28).

The noise level of the quadcopter was 72 dB, and that of the hexacopter was 92 dB. The frequency peaks for the quadcopter were 22.86, 302, 969, 2113, 2551, 3756, and 5884 Hz, and those for the hexacopter were 2985, 82.18, 157, 464, 8800, and 9605 Hz.

At the end of this experiment, the attenuation of the noise emitter (multicopter) was calculated. With the acquired data, it was possible to determine the maximum theoretical audibility emitted from the given source. This calculation was performed for ideal conditions without external noise.

The results show a noticeable difference in noise level between both types of drones. This expected difference was caused by the dissimilar drone construction and the overall output power. The results of this experiment are mainly applicable for echolocation devices. UAV audibility by human senses was evaluated separately. The results of this experiment show effective audio detection by the microphone device at a distance from 70 to 300 m.

#### 4.2.4. Detection by Human Senses

The series of UAV detection experiments using human senses (sight and hearing) in the real environment were performed in these conditions:

- number of evaluators/observers: 6;
- age distribution of evaluators: 21–43 years;
- term of experiment: July and September;
- climate zone and place: temperate, Czech Republic;
- weather and temperature: sunny, 25–30 °C (measured by Extech EN300);
- visibility: 30+ km;
- wind speed: 0–2.5 ms^-1^ (measured by Extech EN300);
- humidity: 45–50% (measured by Extech EN300);
- UAV type: DJI Phantom 2.

**Figure 29:**
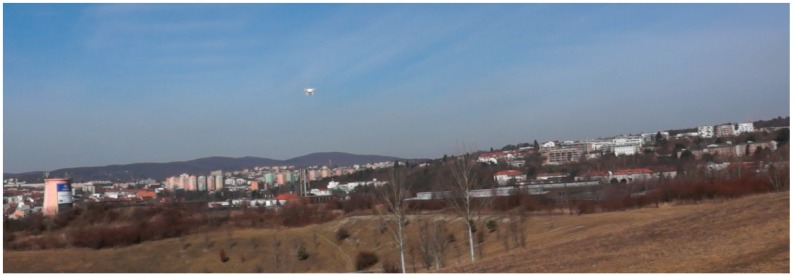
Visibility on the sky background [32]: DJI Phantom 2; distance: 100 m.

General results of visibility by human sight (see Figure 29):

- 100 m: The UAV was visible against a sky background and against an urban (forest) background with minimal effort;
- 200 m: The UAV with no motion was hardly visible against an urban (forest) background; however, during motion with low contrast, the UAV was visible with some effort; against the sky background, the UAV was visible;
- 300 m: The UAV was visible during motion with a high amount of effort against the sky background. With knowledge of the probable position, it was visible against the urban (forest) background;
- 400 m: The UAV was visible during motion with a high amount of effort against the sky background. With knowledge of the probable position, it was visible against the urban (forest) background;
- 500 m: The UAV was visible with a very high amount of effort and with knowledge of the probable position against the sky background (with no clouds);
- >500 m: The UAV was almost invisible during motion. Against the sky background, it was possible to detect the UAV, but persistent tracking was impossible.

**Figure 30:**
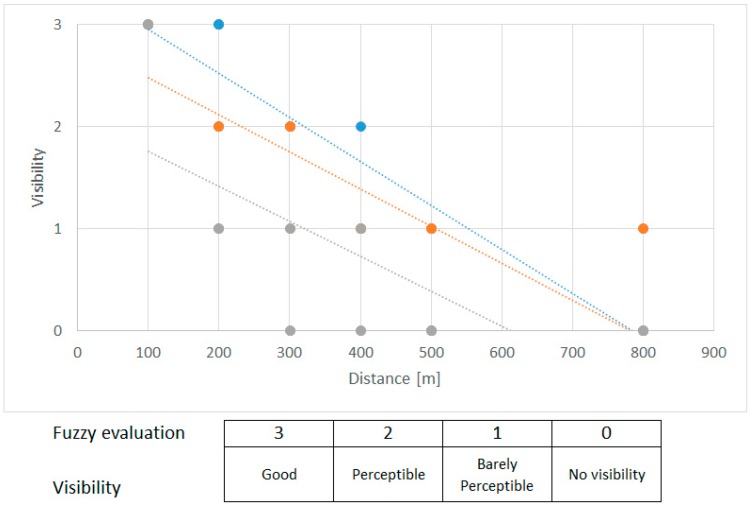
Linear regression of the results of subjective visibility.

Visibility results are graphically presented in Figure 30. The mean value of each observation was processed via fuzzy scoring. Blue represents the visibility against the sky background of the sky, orange stands for the urban (forest) background, and grey represents the visibility on a dry grass background. We concluded that the distance of effective UAV detection by human sight is about 200–300 m, which is very close to the IR detection limits when using commercial IR detection devices.

General results of audibility by human hearing:

The structure of this experiment was simplified: The UAV was flying away from the evaluators, who knew the time and the direction of the flight. In a real situation, the direction of the flight would probably be the reverse. Dependence on the UAV altitude was also evaluated.

- 100 m: The UAV was audible with minimal effort even in noisy conditions (e.g., there was a moving truck at a 200 m distance). It was possible to detect the direction of the UAV by human hearing;
- 200 m: The audibility of the UAV at an altitude up to 3 m was very low and audible with a high amount of effort. At an altitude of about 7 m, audibility was good and the UAV was audible with minimal effort;
- 300 m: At an altitude of up to 15 m, the UAV was almost inaudible. At an altitude of above 15 m, it was possible to detect the direction of the UAV with some effort;
- 400 m: At an altitude of up to 50 m, the UAV was inaudible. At above 50 m, it was possible to hear it with more effort;
- 500 m: At an altitude of up to 50 m, the UAV was inaudible. At above 50 m, it was possible to hear it with a very high amount of effort in the case of minimum background noise.

**Figure 31:**
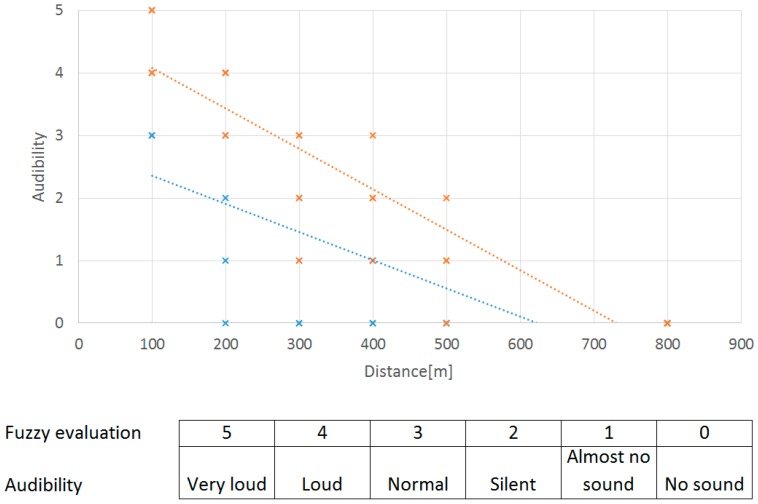
Linear regression of the results of subjective audibility.

The results of audibility are graphically presented in Figure 31. Blue represents audibility during flight close to the ground (7–10 m) and orange represents flight at an altitude of approximately 50–100 m.

The distance limit of effective audibility was about 300 m, which is close to the visibility results (by human sight). However, there is a significant dependence on background noise, which can reduce the sound of a UAV, even at close distances. On the other hand, at night (with minimal background noise), audibility is substantially improved, and the UAV is audible at greater distances.

## 5. Results

The main aim of the study was to verify, clarify, and summarize the real-time detection of commercial drones. Real-time UAV detection is essential for possible counter-action or elimination. Human senses without technical devices are the least effective method of detection. However, in a terminal phase of UAV flight, human senses are usually the main means for weapon aiming and drone elimination.

The first detection sentinel line is usually executed by an X-band radar, which can provide early warnings. The effectivity of this kind of system significantly depends on the skills and training of radar operators’ crew. However, the availability and usage of a radar are limited, and a radar is mostly used by military or air traffic control organizations. We recommend developing such methods of detection for military or ATC users. At present, several studies about those methods have been published, and the research is in progress [33,34,35,36].

The average RCS of the evaluated UAVs was in the range of 0.05 to 0.2 m^2^ (in certain directions, the value of the RCS peak was 0.5–0.75 m^2^). Measured values correlate with values from similar experimental measures [37]. Radars, which are currently in operational use, are usually able to find a UAV (made “scan”), but they are not able to continuously “track” this type of target, especially at longer distances, or can only do so for a limited time, intermittently, with interruptions, etc. The probability of detection therefore depends on the target RCS, terrain configuration, and other aspects.

**Figure 32:**
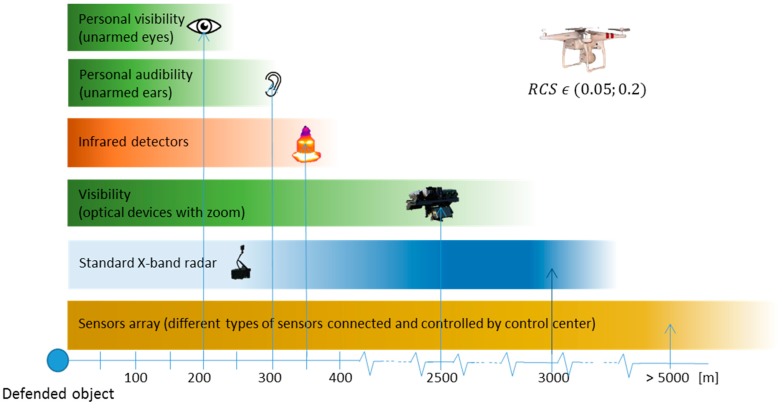
Overview of the results of the performed experiments.

Other evaluated methods of detection have been used in terminal areas of defense and in commercial contexts for security purposes (Figure 32). It is important to develop and improve the methods of detection in IR, visible, and acoustic spectra in connection with algorithms for the autonomous evaluation of acquired data (image, noise, etc.). Such research is now being pursued [38,39,40,41].

## 6. Discussion and Future Directions

This study shows a number of results that help to clarify current methods for UAV detection in real time.

The results of the study show that X-band radars are usually able to detect LSS targets (UAV) at distances of less than 3000 m. However, detection is mostly in the form of radar scans or plots and not radar tracks. Based on these results, we recommend focusing on multispectral detection with a combination of radar, IR, visual, acoustic, radio, etc. Initial detection (scan, plot) is executed by an X-band radar (or a radio receiver), and more precise detection at closer distances is achieved by other methods. Once initial detection and localization of the UAV is achieved, further action can be taken (lethal or non-lethal elimination).

The authors performed several experiments with standard radars (not specialized to detect small RCS objects); however, they do not have a mandate to publish them in this paper. A radar, that is not primarily designated to detect small RCS objects, could be used just as the early warning or alarm device. This early warning may be triggered through the scan or several scans or short track on the radar scope before the track disappears due to the radar threshold parameters. The authors admit that it would not be effective to implement radars, not designed to detect small RCSs, to multi-sensor fields. They also think that producers should work on innovations to cope with such a disadvantage. There are some radars now, for example, C-RAM radar Arthur, which would be suitable for UAV detection when innovated.

Experiments in the IR, visual, and acoustic domains achieved approximately equal detection limits. All of these domains (IR, visual, and acoustic) are appropriate for terminal UAV detection at closer distances (up to 300 m). However, this distance is usually not sufficient for the non-autonomous reaction procedures (a UAV with a flight speed of about 10–15 ms^-1^ can pass this distance in 20–30 s). For this reason, we recommend a C-UAV approach and the implementation of autonomous defense systems with a maximum reaction time of 20 s [42,43].

For the complex defense of crucial objects, a combination of various types of sensors and the corresponding real-time data exchange with a command and control system (C2) or with a surveillance control system is necessary in non-military applications. This system autonomously fuses the data from multiple sensors and evaluates possible threats. The operator (the person in the loop concept) chooses which effectors should be used. In the case of a lack of time or during a high-density attack, effector use can also be decided autonomously, but lethal elimination (weapons) should only be controlled by a human operator (for ethical and safety reasons) to avoid friendly fire or collateral damage.

Research on the C-UAV domain is now focused on active defense against the UAV. There is work on lethal or nonlethal elimination by UAVs [19,44], but this research is still in progress. This topic has been discussed and researched intensively [45,46,47,48]. This reflects recent UAV incidents (attacks). The final step of this research will consist of result fusion (detection and elimination) and implementation in real systems.

Another relatively separate, but more complex, issue, both in terms of detection and defense, is the elimination of group-based UAVs, i.e., swarms. Swarm management and coordination is conditioned by the implementation of highly sophisticated sensor systems and artificial intelligence. In the case of homogeneous or heterogeneous UAV swarms, there are a number of new capabilities and detection methods. The application of active radar methods will depend not only on the characteristics of each individual UAV, but also on the distance between them. That is, if the radar’s capabilities can form a “group target” (where the radar “assumes” that more than one UAV is a single target), then the detectability distance will probably increase. If spacing between UAVs is greater than the resolution of the radar, it will be classified as a “group of targets.” From a detection point of view (in the radio frequency band), it will be possible to benefit from the high density of radio traffic between UAVs in a swarm. Defense against UAV swarms is a sub-division of C-UAS research and will be paid some attention in the future.

## 7. Conclusions

This study covers four years of research and supports the design concept of future defense and protection systems against UAV threats. Together with the development of radars for LSS targets, there is great potential for the development of robust and reliable systems that are able to detect and eliminate UAV threats. The key attribute of the suggested system is its modularity. Potential users (military, air traffic control, security services, etc.) can adjust the system to their needs. However, many conceptual and technical aspects of the C-UAV systems still need to be addressed. Of course, a general solution that reflects all possible situations does not exist—hence the phrase, “there is no silver bullet.”

The hypothesis, confirmed by this study, defines a detection area of up to 2000 m and a terminal area of up to 300 m around a guarded location. These are key areas for sensor network installation and for the use of counter UAS equipment.

The last phase of the study consisted of an analysis of current detection methods of small-sized targets, from mini or micro UAS categories, across the entire alert system. This last step concluded the logical link among the three basic pillars of air defense—sensors, assessment systems (command and control systems), and effectors (lethal or nonlethal engagement against UAS).

## Figures and Tables

**Table 1:** Commercial drone categories.

Category	Range [km]	Max. Altitude [m]	Operational Time [hrs]	Weight [kg]
Nano	<1	100	<1	<0.025
Micro	<10	250	1	<5
Mini	<10	300	<2	<25

**Table 2:** Radar cross section (RCS) experiment results.

Frequency of Radiated Signal [MHz]	RCS of Quadcopter DJI Phantom 2 [m^2^]	RCS of Hexacopter DJI S900 [m^2^]	RCS of Octocopter 3DR X8 [m^2^]
1500	/	/	/
2400	0.01	0.04	0.05
3600	0.12	0.23	0.30
6000	0.11	0.30	0.34
8500	0.26	0.28	0.33
10700	0.10	0.32	0.42

**Table 3:** Distance RF detection and identification.

Position No.	Distance [m]	DJI MAVIC Pro	DJI Ph II	3DR Y6	Note
1	70	A + I	A + I	A + I	
2	220	A + I	A + I	A + I	
3	440	A + I	A + I	A + I	
4	790	A + I	A + I	A + I	
5	1130	A + I	A + I	A + I	
6	1400	A + I*	A + I*	A + I	* at min. altitude 10 m AGL
7	1740	-	A* + I**	A + I	** at min. altitude 20 m AGL
